# Internet-based self-help intervention for procrastination: randomized control group trial protocol

**DOI:** 10.1186/s13063-023-07112-7

**Published:** 2023-02-06

**Authors:** Ying Zhou, Jianhua Wang

**Affiliations:** 1 School of Education, Beijing Open University, Beijing, 100081 People’s Republic of China; 2 School of Foreign Languages, Renmin University of China, Beijing, 100872 People’s Republic of China

**Keywords:** Procrastination, Internet-based intervention, Control group randomized trial, Self-regulated learning theory, Cognitive behavioural therapy

## Abstract

**Background:**

Procrastination or “postponing until later” is a common phenomenon defined as the intentional delay in partaking in and finishing important activities despite negative outcomes potentially outweighing the positive. Procrastination adversely affects mental health, academic performance, and career achievement. Although studies on procrastination intervention methods and effectiveness exist, utility and cost-effectiveness are limited by various factors, including practitioner availability and skills, barriers to participant participation, and the time investment required by participants. Thus, internet-based interventions could increase the availability of evidence-based treatments for adult procrastination.

**Methods:**

This study explored the efficacy of an online-based self-help intervention in the context of voluntary procrastination among undergraduate psychology students. The study design is a randomized controlled trial. Participants who self-reported procrastination-related problems and behaviours were included in the trial consisting of two groups; specifically, one group undergoing a self-directed internet-based intervention for coping with procrastination (*N*=160) and (2) another group with delayed access to the intervention programmes (waitlist control group; *N*=160). Follow-up assessments were scheduled 6 and 12 weeks after baseline, and the control group received the intervention after 12 weeks. Procrastination, measured by the Irrational Procrastination Scale and the Simple Procrastination Scale, was examined as the primary outcome. Meanwhile, secondary outcomes included susceptibility, stress, depression, anxiety, well-being, self-efficacy, time management strategies, self-control, cognition, and emotion regulation. Other measures comprised acceptability (e.g., intervention satisfaction, potential side effects, and expectations) and learning behaviour analysis to reflect adherence.

**Discussion:**

This randomized controlled trial will provide data on the effectiveness of online interventions for adult procrastination. If deemed effective, this low-cost, high-coverage internet-based intervention could aid more people who seek to address their procrastination.

**Trial registration:**

Chinese Clinical Trial Registry. https://www.chictr.org.cn/showproj.aspx?proj=171246.

## Strengths and limitations

1. Applying internet-based self-help interventions may help treat those with active procrastination.
2. The protocol involved weekly questionnaires, increasing the external validity and time resolution.
3. The effects of the interventions were assessed by examining the changes in self-reported and objectively measured procrastination behaviours.
4. Revealing the possible mechanisms of various changes helps advance the precision of procrastination interventions.
5. The large number of participants and high dropout rate may have affected the outcomes.

## Background

Procrastination or “postponing until later” is a common phenomenon defined as the intentional delay in partaking in and finishing important activities despite the negative outcomes potentially outweighing the positive. One characteristic of procrastination is the failure to self-regulate [1], involving problems related to all three stages of self-regulation [2]. While the pre-action phase is associated with the loss of self-determination to accomplish tasks, the action phase involves issues concerning concentration and blocking distractions [3]. The post-action phase is associated with low self-efficacy [4]. Academic procrastination is linked to inadequate cognitive and metacognitive learning strategies [5]. The second characteristic is emotional discomfort. Unlike strategic delay, procrastination can be accompanied by subjective discomforts, such as fear of failure [6], emotional burden [7], guilt, and shyness [8]. Third, cultural differences exist in evaluating delay, particularly delayed tolerance or evaluation subject to internal norms [9]. The degree of acceptable delay also varies by culture [10].

Procrastination is common in online learning [11], everyday life [12], and the workplace [13]. The literature [14] indicates that 80–95% of college students procrastinate, and approximately 15–25% of adults report chronic procrastination. Although problematic procrastination is not a clinical diagnosis in either the latest version of the Diagnostic and Statistical Manual or the International Statistical Classification of Disease and Related Health Problems, however, it can be problematic behaviour if it becomes routine and causes distress. Besides, procrastination is often thought of as a symptom commonly found in various disorders. So we need to address that problematic behaviour of gross lack of punctuality.

It is vital to provide effective treatment for people who engage in procrastination as it can result in a range of negative consequences, such as decreased learning [15], hindered career development [16], and impaired physical and mental health [17]. Overcoming procrastination is the focus of research, practice workers and procrastinators.

Regarding the treatment of or intervention to curb procrastination, some researchers have evaluated the effectiveness of relevant treatments through reviews [18] or meta-analyses [19, 20]. These studies concluded that people’s procrastination behaviours can be changed; thus, procrastination interventions are essential for students during the learning phase. Self-regulation is perceived as a crucial trait in achieving academic goals. The follow-up study revealed that the behavioural tendencies of the subjects did not rebound or recur after the procrastination intervention, and its effect remained stable. However, the feasibility and cost-effectiveness of these interventions were not high because the research was influenced by several factors, such as accessibility, practitioner skills, the barriers faced by participants, and the significant time commitment required by participants [20, 21].

Digital procrastination interventions delivered online on a computer or mobile device could be a promising alternative to overcome the above barrier [22, 23]. In addressing procrastination, online interventions have several advantages over traditional face-to-face therapy. First, adaptively publishing training or relevant information tailors the interventions to the individual needs of the subjects. Second, costs for both the instructor and the subject can be reduced by improving the treatment efficiency [24]. Moreover, internet-based intervention channels for procrastination include online counselling and psychotherapy [25], interactive and self-guided intervention [25], online support groups [26, 27], online virtual reality therapy [28], gaming therapy [29], mobile internet and short message service texting [23, 30], applications [31–33], video chat and conferences [34], and email [35].

One study revealed tentative signs that internet-based interventions for procrastination may be acceptable [30, 33, 35, 36]. During the COVID-19 pandemic over the past 2 years, particularly in low-and middle-income countries, people experienced prolonged physical distancing and reduced face-to-face social interaction. This effect resulted in considerable challenges to mental health service systems worldwide [37]. Changes in learning environments have led people to procrastinate their tasks more readily, especially student groups, reducing learning engagement and increasing the tendency to procrastinate [38]. The UN and various experts have recommended digital psychological interventions for mental health issues, given their potential to provide the necessary psychological support.

However, evidence on the efficacy of online psychological interventions on procrastination has been erratic and insufficient. For example, there is only limited research on online psychological interventions for procrastination, especially in developing countries. According to research outcomes, most intervention programmes are not based on theoretical frameworks. From the research design perspective, the number of randomized control trials (RCTs) is limited, and the quantitative diagnosis of procrastination differs, reducing the comparability of studies. In addition, it is often impossible to execute follow-up studies due to the withdrawal of participants and an overly short intervention period. The results of some studies have highlighted that counselling via online chat and face-to-face consults has the same effect on procrastination [25], while self-guided online intervention groups demonstrate regression during follow-up monitoring [39].

There are many current theories on procrastination behaviour intervention with incomplete statistics, including the Social Cognition Theory [40], the Theory of Planned Behavior [41], the Goal Setting Theory [42], and the Self-Determination Theory [43]. The four internet-based psychological intervention methods are motivation enhancement, cognitive behavioural therapy (CBT), community reinforcement methods, and emergency management. The three procrastination intervention methods [20, 44] are self-regulation (e.g., time management and emotion regulation training), CBT, and other therapies (e.g., conflict intervention, coherence therapy, and Acceptance and Commitment Therapy) focused on personal strengths and resources. These interventions include psychoeducation, social support, and relaxation techniques.

The first internet-based intervention to reduce procrastination was created by Rozental [45]. In contrast, Eckert et al.’s 30 interventions involving text messaging concentrated more on approaches that adaptively address emotional disorders indicative of procrastination, particularly those that enhance tolerance and change emotionally aversive tendencies.

This study integrates aspects of the three stages of CBT, including irrational cognitive correction, self-regulation learning theory, emotion regulation skills, and time management strategies. A targeted intervention programme was developed involving a limited volume of reading and homework that takes place within a sufficient period of time. In this manner, this study adds to the literature regarding online-based interventions for procrastination. Additionally, our approach integrates self-regulation theory, time motivation theory, and CBT to execute interventions through the internet and investigate the effect of psychological behavioural intervention on online learning-related procrastination.

This study primarily examines the impacts of a self-motivated online-based psychological intervention on (a) procrastination behaviour and (b) impulsivity, depression, anxiety, well-being, self-efficacy, time management strategies, self-control, and cognitive emotion regulation. The outcomes were compared with those of the control group. Then, in the context of the intervention, the experience and adherence of the users were investigated.

## Methods/design

**Fig. 1:**
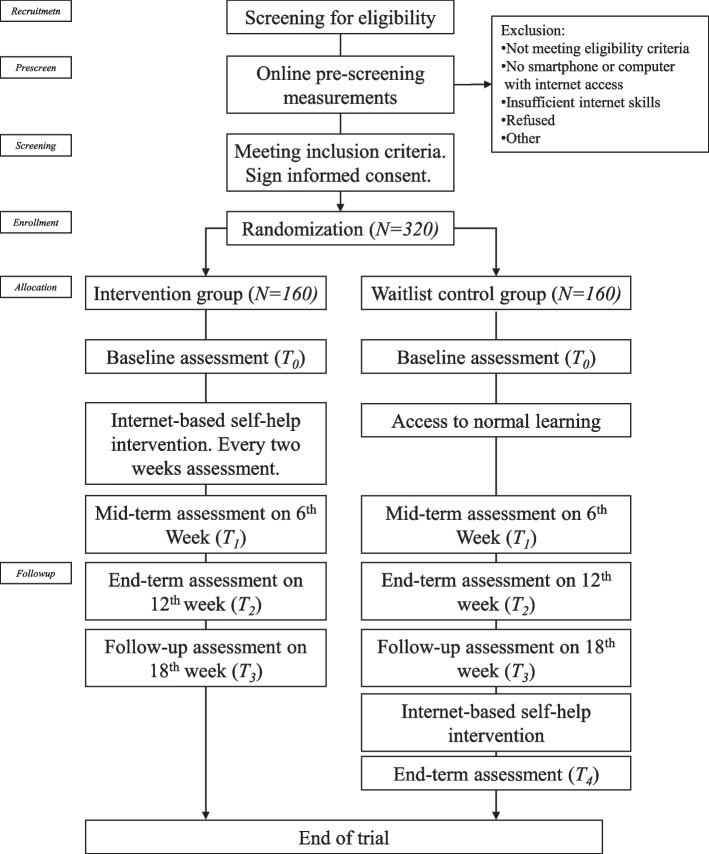
CONSORT flow diagram of the study participants

This paper utilized a randomized control trial (RCT) design involving a group that underwent self-guided psychological intervention delivered online and a waitlist control group that did not receive the intervention. Figure 1 illustrates the CONSORT flow chart for this study. We hypothesized that the outcomes of those undergoing psychological intervention would be superior to the outcomes of those in the control group. The study consisted of two follow-up assessments and a follow-up assessment at 6 (T1), 12 (T2), and 18 (T3) weeks after the baseline measurements were taken that could also be used after a self-directed intervention plan. Another assessment (T4) of the control group was performed for an initial assessment of the effect.

### Setting and recruitment

The participants were all psychology students, and recruitment took place from January to February 2023. The students received a link to the electronic questionnaire, which included a detailed study description and trial registration information. Each participant was invited to sign an informed consent form. Then, upon completing the entirety of the study, a certain number of credits or gift vouchers were given to the eligible participants in tune for their participation.

### Eligibility criteria

The primary criterion for the participant’s eligibility was the person’s self-reported Irrational Procrastination Scale (IPS) score; specifically, a score equal to or greater than 32. Before randomization, the participants were screened based on the following exclusion and inclusion criteria:

Inclusion criteria:

1. Aged above 18 years.
2. Access to and ability to use computers, smartphones, and the internet.
3. Ability to speak, write, and read Chinese.
4. Enrolled in study at a university or college.

Exclusion criteria:

1. Current self-reported diagnosis of at least one serious mental illness.
2. Continued drug abuse.
3. Continued participation in another form of psychotherapy.

### Withdrawal

Eligible study participants assessed by the screening questionnaire were required to digitally sign an informed consent form (Appendix). They could withdraw their participation on any grounds at any time. Additionally, if a participant’s condition deteriorated, the study supervisors could end their participation earlier than scheduled and direct them to receive official healthcare.

### Randomization and blinding

The random number generator functionality in Microsoft Excel 2007 was used for randomization. The randomization results were automatically conveyed to the participants. Their classification into the intervention and control groups was not explicitly communicated. Additionally, for the intervention, the participants were informed that the waiting period varied between 0 and 12 weeks at random. After randomization, the participants could immediately access the link (via email) to the intervention or the relevant information stating that the intervention would commence after 18 weeks. We do not anticipate any requirement for unblinding, but if required, the Trial Manager will have access to group allocations and any unblinding will be reported.

Group tasks were masked throughout the data analysis to reduce potential statistical method-related biases. All information on the dataset specifying the group assignments was deleted by the independent researchers.

### Control condition

Individuals assigned to the waitlist control group did not have access to the online-based intervention during the 12-week assessment period. After completing their final assessment, they received an email with an access link for the intervention. Throughout the study period, participants in both groups could receive other treatments, and such treatments were measured at every evaluation.

### Intervention

The internet-based intervention was a 12-week self-help programme based on time motivation theory and self-regulation learning models. It adopted methods and exercises related to time management, emotion regulation, and CBT. The contents of the intervention are presented in Table 1.

**Table 1:** Contents of the 12-week internet-based intervention for procrastination

Module	Content	Homework
1. General introduction	Self-regulated learning, academic procrastination and other theories, self-regulated learning strategies.	Develop overall learning goals, understand deadlines, and set one study day per week.
2. Goal and time management	Set the purpose and content of the subgoals, train to prepare a to-do list before the study day and record the work progress during the study day.	Set and share subgoals, timelines, and checklists.
3. Manage unreasonable thoughts	Employ behavioural activation techniques, self-esteem strategies, and strengths-finding to encourage participants to see things positively.	Record success stories, personal abilities, and strengths.
4. Emotion regulation	Introduce the meaning of negative emotions, cognition, mindfulness and other methods to regulate negative emotions, and share video files for relaxation.	Mindfulness and relaxation exercises.
5. Self-motivation	Offer two self-motivation strategies: managing the learning environment and adopting self-reinforcement. Introduce work environment and study table design, self-reinforcing principles and benefits.	Optimize the learning environment and take photos to share; develop self-reinforcing strategies around each of your subgoals.
6. Distractions and temptations	Self-directed strategies are introduced to manage interference and temptation to guide students to make plans managing such scenarios in advance.	Complete the cognitive distraction “if...then...” sentence.
7. Prepare for the future	Aware of the inevitability of procrastination again and be prepared to deal with it	Organize the course resource catalogue.

The online Moodle platform was accessed by the participants on their computer or mobile smart device. After registration, all modules were available free for 6 months, but the programme recommended completing courses in the intended sequence over a 12-week period. Each participant studied the course content independently. The integrated messenger feature was available for the participants to receive technical support and inquire about the exercises. Additionally, psychologists tracked the participants’ progress and monitored adverse developments. Viewers who did not log in in after receiving the access link received an email reminder.

### Measures

The results included questionnaires and learning behaviour data. Assessments were repeated at baseline, 6 weeks, and 12 weeks. Table 2 provides an overview of the measurements including the schedule of measures and the timelines.

**Table 2:** Schedule of measures and timelines

	Enrolment	Baseline	Every 2 weeks (only intervention group on)	Mid-treatment	Post-treatment
Enrolment - Eligibility screen - Informed consent	×				
Demographic variables - Gender - Age - Education - Marriage - Courses - Semester - Psychotherapy experience - Income - Job - Email - Phone number	×				
*Primary confirmatory outcome*					
Procrastination (IPS)	×		×	×	×
Procrastination (PPS)		×	×	×	×
*Secondary confirmatory outcome*					
Depression (PHQ-9)		×	×	×	×
Anxiety (GAD-7)		×	×	×	×
Stress (PSS-10)		×	×	×	×
Well-being (WHO-5)		×	×	×	×
*Secondary exploratory outcome*					
Learning self-efficacy (SSE)					×
Self-control (SCS)					×
Cognitive emotion regulation (CERQ)					×
Time management strategy (MSLQ-B)					×
*Other measures*					
Attitudes towards online intervention (APOI)				×	×
Risk and side effects (NEO)				×	×
Credibility (CEQ)				×	×
Satisfaction				×	×
Special time period - Taking an exam - On vacation				×	×
Changes in other treatments				×	×
Usability (module understanding, use, benefit)			×		
User learning behaviour data - User login - Complete the module - Complete the questionnaire - Finish homework - Complete the event - Time to read text - Time to practice - Number of clicks			×	×	×

*Note: IPS* Irrational Procrastination Scale, *PPS* Pure Procrastination Scale, *PHQ-9* Patient Health Questionnaire-9, *GAD-7* General Anxiety Disorder-7, *PSS-10* Perceived Stress Scale (PSS-10), *WHO-5* The World Health Organization-Five Well-Being Index, *SSE* Student Self-efficacy Scale, *SCS* Self-Control Scale, *CERQ* Cognitive Emotion Regulation Questionnaire, *MSLQ-B* Motivated Strategies for Learning Questionnaire part-B for distance learning, *APOI* Attitudes towards Psychological Online Interventions, *NEO* Negative Effects Questionnaire, *CEQ* Credibility/Expectancy Questionnaire

### Primary confirmatory outcome

1. Procrastination (Irrational Procrastination Scale, IPS)

Procrastination was evaluated using the Chinese version of the Irrational Procrastination Scale [46], which included 9 items on a Likert 5-point scale, ranging from 1 to 5, indicating “strongly disagree” to “strongly agree”. The internal consistency coefficient of the scale was 0.73 [47].

(2) The Pure Procrastination Scale (PPS) measures the general level of one’s procrastination in various subjects. The Chinese version of the scale [48] includes 12 items divided into two dimensions: the procrastination process and overdue tasks. The Likert 5-point scale was used to score from 1 to 5, indicating “strongly disagree” to “strongly agree”. The higher the score is, the higher the level of procrastination caused by the individual’s ego dysfunction will be. Cronbach’s alpha coefficient was 0.85 [49].

### Secondary exploratory outcome

1. Depression symptoms (Patient Health Questionnaire-9, PHQ-9)

To examine the depressive state of the participants, the Chinese version [50] of the PHQ-9 was used, including 9 items. The questions focused on the frequency with which the participants experienced specific symptoms in the past 2 weeks and were scored using a Likert 4-point scale. The range of the total score was 0 to 27 points. Higher scores indicated greater depression severity, comprising depressive symptoms that were marked as severe (20–27), moderate to severe (15–19), moderate (10–14), mild (5–9), and absent (0–4). Teymoori et al. [51] marked the presence of depression with a PHQ-9 score ≥5, and 0.82 was the internal consistency coefficient of the questionnaire.

(2) Anxiety symptoms (General Anxiety Disorder-7, GAD-7)

The anxiety level of the participants was assessed using the Chinese version of the GAD-7 scale [52], which included 7 items. The questions explored the frequency with which the participants experienced anxiety over the past 2 weeks, scored using a Likert 4-point scale ranging from 0 to 3, indicating “nothing at all,” “a few days,” “more than half a day,” and “almost every day.” The total score that evaluated the symptoms of anxiety ranged from 0 to 21 points; specifically, none (0–4), mild (5–9), moderate (10–14), and severe (15–21). A GAD-7 score ≥5 indicates the presence of anxiety disorder and its symptoms, with an internal consistency coefficient of 0.89 for this scale [53].

(3) Perceived stress (Perceived Stress Scale, PSS-10)

The PPS-10 was used to quantify the level of stress and control perceived by the subjects in the previous month [54]. The Chinese version of the PPS-1054 includes 10 items (6 negative items [1–3, 8, 11, 14, and] and 4 positive items [6, 7, 9, 10, and]). A Likert 5-point scale from 0 to 4 indicating “never” to “very frequent” was used, and the score ranged from 0 to 40. A higher total score for all items (after the negative scoring of positive items) signified greater stress and perceived loss of control. The internal consistency coefficient of the scale was 0.75 [55].

(4) Index on well-being (WHO-5 or World Health Organization-Five Questionnaire)

This questionnaire was utilized to investigate the relevant changes in the context of the participants’ overall well-being. The 5 items were scored using a 6-point Likert scale ranging from 0 to 5, indicating “no time” to “all the time.” A total score below 13 denoted poor levels of mental health, which was an indicator of depression. The internal consistency coefficient was 0.92.

### Secondary confirmatory outcome

1. Self-efficacy in learning (Student Self-efficacy Scale, SSE)

The SSE was used to gauge the students’ confidence in their capacity to realize their anticipated academic performance. The Chinese version [56] had a total of 10 items scored on a 4-point Likert scale ranging from 1 to 4, indicating “completely incorrect” to “completely correct.” The internal consistency coefficient was 0.66.

(2) Self-control (Self-Control Scale, SCS)

The SCS was utilized to assess participants’ self-control and regulation abilities. The Chinese version [57] had 19 items and used a 5-point Likert scale to score, ranging from 1 to 5, indicating “very unsatisfactory” to “very agreeable”. The five dimensions were impulse control, healthy habits, resistance to temptation, dedication, and entertainment moderation. The higher the total score was, the stronger the self-regulation control ability was. The internal consistency coefficient was 0.84.

(3) Cognitive and emotional regulation strategy (Cognitive Emotion Regulation Questionnaire, CERQ)

The CERQ was employed to evaluate the specific cognitive strategies that individuals use when faced with negative events. The Chinese version [58] included 36 items and used a 5-point Likert scale to score, ranging from 1 to 5, indicating “never” to “always”. Each of the 9 dimensions comprised 4 items; the 9 dimensions were self-blame, acceptance, contemplation, positive refocusing, refocusing on planning, positive re-evaluation, rational analysis, catastrophic, and blaming others. A higher score on a dimension indicated a greater likelihood of using that particular cognitive strategy when faced with a negative event regarding time.

(4) Time management strategy for adult distance learning (Motivated Strategies for Learning Questionnaire Part-B for distance learning, MSLQ-B)

The time management strategies subscale in the Chinese version of the Distance Learning Motivational Strategies Questionnaire [59] was used. The subscale included 3 items scored on a 6-point Likert scale, ranging from 1 to 6, denoting “very inappropriate” to “very appropriate” or “agree” to “strongly agree”. Higher scores indicated better application of the learning strategy.

### Other measures

1. Attitude towards online psychological intervention (Attitudes Towards Psychological Online Interventions Scale, APOI) [60]

The APOI questionnaire was used to determine the participants’ concerns regarding the adverse events, feasibility, professionalism, and effectiveness of online psychological treatments or interventions. More specifically, the APOI comprised 16 items categorized into four subscales, “confidence in effectiveness”, “doubt and risk perception”, “benefit of anonymity”, and “technology threat”. The subscales were scored on a 5-point Likert scale ranging from 1 to 5, specifying “completely disagree” to “completely agree.” Stronger levels of positive attitudes were denoted by higher “benefit of anonymity” and “confidence in effectiveness” subscale scores. Meanwhile, stronger levels of negative attitudes were noted with higher scores in “technological threats” and “doubt and risk perception.” The internal consistency coefficient of the questionnaire was 0.77 (0.62, 0.72, 0.64, and 0.62 for the four subscales, respectively).

(2) Negative effects of the treatment (Negative Effects Questionnaire, NEO) [61]

The 20-item NEO was adopted to identify the adverse effects of the interventions. The respondents were interviewed about the occurrence of an adverse event (yes/no), the strength or extent of the effect (0 to 4), and whether the side effect was attributable to the intervention or other causes. Accordingly, two scores were acquired: the extent of the adverse effects (0 to 80) and the frequency of treatment-related reactions (0 to 20). The internal consistency coefficient of the questionnaire was 0.95.

(3) Credibility and expectancy (Credibility/Expectancy Questionnaire, CEQ) [62]

The credibility subscale assessed beliefs about the treatment’s effects, while the expectation subscale evaluated the extent to which the participants felt that their symptoms improved during the intervention. This 6-item questionnaire had an internal consistency coefficient of 0.84.

(4) Satisfaction

At the end of every module, the following four open-ended questions were asked to measure user satisfaction:

- Were some parts helpful or reassuring?
- Did some parts make you feel worthless or evoke complex emotions?
- For family members or friends experiencing challenges similar to yours, would you recommend this module?
- On a scale of 1 to 10, how valuable was this module?

(5) Compliance

The learning behaviour profile on the Moodle platform was used to measure patient compliance, including the number and dates of participant logins to the online intervention, the number of modules completed, the number of exercises completed, and the total time expended on the modules and exercises. In the study assessment, 6 and 12 weeks later, the intervention group participants were interviewed about their frequency of use of the intervention.

## Safety

Based on the findings of similar studies, we anticipate minimum risk of adverse events and serious adverse events in the study. When the participants would report the negative effects, the author will help them to get help from professional advisors. All adverse events will be treated, recorded in detail, and reported to the Research Ethics Committee immediately.

## Data monitoring

Data will be collected and managed by a researcher. All the data will be stored in hard drives. The Ethics Committee who is independent from the sponsor and competing interests will supervise this trial and will make the final decision to terminate the trial. The project management group will meet every month.

## Data analysis

The four-part statistical analysis of this paper comprises (a) descriptive statistics, (b) the main outcome’s confirmatory analysis with a sensitivity analysis, (c) secondary outcomes and their assessment with a sensitivity analysis, and (d) an adjustment effects analysis. All statistical analyses were performed using SPSS.

### Primary and secondary outcome analyses

The clinical and sociodemographic variables of both groups were analysed at baseline using descriptive statistics. The impacts of the online intervention on procrastination were individually assessed for each measure. A repeated-measures linear mixed-effects model was utilized. With repeated measures of the same subjects over time, the model was appropriate for longitudinal data analysis, particularly for the missing data. Cohen’s *d* statistic was employed to calculate the size of the treatment effect between and within the groups, with the critical values being 0.8, 0.5, and 0.2 when the effect was small, medium, and large, respectively. Then, an analysis was performed to estimate the number of participants who attained clinically significant changes at the end of the treatment and during the follow-up. The pre-intervention IPS scores were compared with the post-intervention and follow-up IPS scores.

### Missing data and sensitivity analyses

Both intention-to-treat and completer analyses were conducted. Completers may be more likely than non-completers. Since there were systematic differences between the subjects, two sensitivity analyses were performed. The first was the conservative last-measures ahead of time (LOCF) method that used the last measurement obtained by each subject, and the second was the chain equation multiple imputation method.

### Additional analyses

The additional analyses included the assessment of potential moderators or confounding variables. Therefore, potential pre-intervention differences in IPS were examined along with the demographic variables between the experimental and control groups by performing independent samples *t* tests. For the intervention × time interaction, these variables were included as moderators to verify the stability of the findings when the two groups were significantly different. The primary outcome could have been affected by the covariates, including emotion regulation, the participant’s stance regarding internet-based psychological interventions, patient satisfaction and adherence, and expected patient outcomes, which were added to the analysis.

Additionally, weekly assessments of procrastination were analysed, allowing us to measure changes in procrastination with higher ecological validity and temporal resolution. In the intervention group, we investigated the percentage of side effects encountered due to the treatment and calculated the degree of damage resulting from these effects. Moreover, the subjects’ self-control, self-efficacy, and time management strategies in their daily lives may be potential moderators or mediators of procrastination reduction.

### Interim analyses

We do not make pre-planned interim analyses. The Ethics Committee will review emerging trial data and external evidence and recommend early stopping.

*Statistical power and sample size*

The sample size was estimated based on the meta-analysis evidence of the effectiveness of the online intervention on procrastination, and the intervention had a moderate effect (−0.58). The sample size was 320; a statistical significance level of 0.05, an average of 4.96 in the experimental group, an average of 2.96 in the control group, and a standard deviation of 5.5 was required. Further effect size analyses, including potential discontinuations, various effect sizes, and within-group correlations, demonstrated that the sample size was sufficiently large to detect moderate effects under different assumptions.

## Patient and public involvement

This trial did not consider patient and public involvement. Patients were not invited to interpret the results and write or edit the document.

## Discussion

Preliminary indications from several studies have revealed that internet-based interventions targeting procrastination are acceptable [3]. However, few relevant evidence-based studies have been conducted, with inconsistent quantification methods for procrastination and limited long-term follow-up studies. Further research on the efficacy of internet-based interventions for procrastination is necessary. The research findings in this paper are expected to contribute to and advance the literature on online-based psychological interventions. Equally important, the results have crucial implications for the therapeutic management of procrastination among adults. The findings presented are also anticipated to mitigate the severity of procrastination among the subjects and improve their quality of life.

This RCT was designed to investigate the efficacy of a 12-week online-based intervention on procrastination in adults. The primary and secondary outcomes of this study compared the intervention and alternate control groups in the context of their respective levels of procrastination. This approach adds high-quality, sufficiently robust evidence to the weak research base for CBT-and internet-based procrastination interventions. We will also study the acceptability of the intervention design, including potential adverse effects and risks, adherence, and intervention satisfaction, to further refine the intervention design. Additionally, we will explore potential moderators and mediators to understand how the intervention works and who might be more valuable.

### Strengths and limitations of this study

With the aim of maximizing the internal validity of our findings, the design of this study as an RCT with a waitlist control group was based on our objective to assess the efficacy of online-based interventions. Our randomization approach abides by the principle of sequential allocation concealment to further lessen the systematic bias. In addition, those who procrastinated on medication or psychotherapy at baseline were excluded to minimize systematic pre-treatment differences.

In addition to investigating the changes in procrastination-related behaviours, we examined how participants benefited from online-based interventions in their daily lives. Since procrastination is linked to a broad spectrum of adverse outcomes, evaluating the changes in one’s work ability, well-being, and psychosocial functioning will aid in assessing online-based interventions from a holistic perspective. Additionally, our study investigated the possible mechanisms of various changes, such as changes in self-esteem, time management, and self-control, which may help advance the precision of procrastination interventions in the future.

However, these advantages may also come with some limitations. First, to achieve the power of statistical analysis, a large number of participants was required, and recruiting such high numbers of participants can be challenging because the proportion of the general population with severe procrastination behaviours is relatively low, as is the number of those seeking help. Certain barriers could limit the number of interested and eligible participants. We used various recruitment strategies, such as offline and online advertising, and allowed participation independent of the subjects’ location.

The second challenges are the study’s high discontinuation rate and low adherence. The latter challenge is particularly concerning in our case because a considerable number of evaluations that merge online-based interventions with standard questionnaires will yield relatively high participation rates among the patients. We provided reminders via phone calls, emails, and text messages to improve compliance. Moreover, our assessment scales were kept as short as possible, incorporating subscales only when necessary. An intention-to-treat method was adopted, and sensitivity analysis was performed for the data analysis to measure the impact of potential dropouts.

The third challenge is the response burden. It may be also problematic in participants with severe procrastination. After the pilot study, we may reduce the overlap of content between instruments in order to reduce response burden.

This research contributes to the growing field of internet-based intervention. First, our trial aimed to assess whether an online psychological intervention could help reduce procrastination and improve psychosocial functioning. Second, our study will augment our knowledge of potential predictors and mechanisms of change in treatment outcomes, enabling us to advance our understanding of how online-based self-intervention could lessen the burden on procrastinators. Third, instead of only concentrating on procrastination-related changes, we included a wide range of evaluation methods and various outcomes. Thus, we could delve deeper into how and whether the participants’ daily lives are affected by online-based interventions.

Based on these innovations, our internet-based intervention for procrastination will provide an evidence-based, low-threshold approach for helping many students who experience procrastination-related problems, reducing social costs, and saving professional resources.

## Trial status

This trial was registered in the Chinese Clinical Trial Registry (https://www.chictr.org.cn) on 14 December 2022 (registered number: ChiCTR2200065752, the protocol version number: V2.0). This study is currently in the recruitment stage. Recruitment will be approximately completed before 28 February 2023, and the trial is estimated to end in 1 January 2024.
